# How Upper/Middle Managers' Ethical Leadership Activates Employee Ethical Behavior? The Role of Organizational Justice Perceptions Among Employees

**DOI:** 10.3389/fpsyg.2021.652471

**Published:** 2021-03-16

**Authors:** Hussam Al Halbusi, Pablo Ruiz-Palomino, Pedro Jimenez-Estevez, Santiago Gutiérrez-Broncano

**Affiliations:** ^1^Sultan Qaboos University, Muscat, Oman; ^2^Faculty of Social Sciences, University of Castilla-La Mancha, Cuenca, Spain; ^3^Faculty of Social and Legal Sciences, University of Castilla-La Mancha, Toledo, Spain; ^4^Faculty of Social Sciences, University of Castilla-La Mancha, Talavera, Spain

**Keywords:** ethical leadership, senior management, ethical behavior, organizational justice, justice dimensions

## Abstract

Several studies have been conducted on ethical leadership and workplace ethical behavior but little is known about the role of organizational justice and each of its dimensions (procedural, distributive, interpersonal, informational) in this relationship. This study predicts that ethical leadership enhances organizational justice perceptions, including each of its specific dimensions, which in turn enhances employee ethical behavior. The results from two-wave survey data obtained from 270 employees in the Malaysian manufacturing industry confirm that ethical leadership has a positive impact on employee ethical behavior, and that organizational justice and each of its justice dimensions mediate this relationship, both individually and together. Importantly, interpersonal and informational dimensions show the strongest mediation effects. This paper highlights the actions and strategies that can help managers to effectively elevate the moral tone in their organizations. In particular, our findings show where managers must put more emphasis to foster an ethical workplace: on providing fair treatment (interpersonal justice) and honest information (informational justice).

## Introduction

Numerous corporate scandals (e.g., Volkswagen, the London Interbank Offered Rate) have occurred in recent years, at causing great harm to society (Al Halbusi et al., 2019, 2020b; Babalola et al., 2019) and to organizations (Padilla et al., 2007). As a result, there is growing interest in understanding the sources of influence of unethical behavior in organizations, raising the need to implement measures to produce more ethical and humane businesses. When people lack ethical behavior, people set out to fulfill their own self-interest above the interests of others (Padilla et al., 2007), which may pose serious problems for organizations (e.g., theft, sabotage, bullying, lying, moral harassment). As such, how to favor ethical behavior is a pressing issue that needs to be studied (Treviño et al., 2014).

Employee ethical behaviors refer to actions that contribute positively to corporate social and ethical performance and require strong guidance and clear value structures within the organization (Brown and Treviño, 2014). Managers' leadership offers clear guidance in this regard. Ethical leadership means that ethical standards are more likely to be perceived by employees, which should foster ethical behaviors (Brown and Treviño, 2006; Mayer et al., 2009). By exercising the highest ethical standards in their day-to-day activities, leaders become role models (Ruiz-Palomino and Linuesa-Langreo, 2018) who are likely to enhance their subordinates' ethical behavior.

Ethical leadership is likely to become critical in terms of enhancing employee ethical behavior; how this happens is a field that needs further development. This relationship could be mediated by other organizational mechanisms. While an ethical climate has been addressed as the best mechanism underlying the ethical leadership–employee ethical behavior relationship (Schminke et al., 2005), ethical leadership could shape other aspects that play an important role in this relationship. In addition to providing ethical guidance, ethical leaders are fair (Ko et al., 2018; Metwally et al., 2019), which entails important aspects such as transparency, balanced decision-making, and giving fair and equal treatment to others (Metwally et al., 2019). Given that leaders are perceived as representatives of the organization (Hou et al., 2018), especially if they occupy upper or middle management positions, the practice of ethical leadership should make employees perceive their organization as fair; in turn, according to social exchange theory (SET, Blau, 1964), employees may become eager to reciprocate (to the leader and the organization that the leader represents) with positive, valuable behaviors (Ko et al., 2018), such as ethical behavior.

If managers are found to behave unethically, followers might question whether organizational rules and guidelines can be relied upon (i.e., organizational injustice, Premeaux, 2009; Xu et al., 2016), which is likely to have a negative impact on employee ethical behavior (Colquitt et al., 2001). However, to our knowledge, existing research has practically avoided the issue of whether organizational justice and its different dimensions could mediate in the relationship between the ethical leadership of senior managers and employee ethical behavior. Previous research has reported social exchange processes as an important underlying mechanism explaining how ethical leaders can encourage positive outcomes (e.g., prosocial behavior, Brown and Treviño, 2006). Social exchange processes have also been observed to be involved in the positive attitudes and behaviors of employees that result from contexts where organizational justice is perceived (El Akremi et al., 2010; Wang et al., 2017). However, little research focuses on whether organizational justice could mediate between the ethical leadership of senior managers and employee ethical behavior; far less research is available addressing which traditional justice dimension (i.e., distributive, procedural, interpersonal, informational) is the most relevant for capturing this mediating effect. Thus, the literature in this area could be advanced by investigating the role of organizational justice and of each of its distinct dimensions in the relationship between the ethical leadership of senior managers and employee ethical behavior.

Our principal research objective is to explicate the role of organizational justice and its most recognized dimensions in the positive relationship between ethical leadership of senior managers and employee ethical behavior. To this end, we first examine the positive effect of senior manager's ethical leadership on employee ethical behavior. Then, we investigate the mediating effect of organizational justice and each of its dimensions to highlight which justice dimension is more relevant in accounting for such an effect. This is an important contribution since, to our knowledge, there is still little research on whether ethical leadership of senior managers fosters all justice dimensions equally and on which justice dimensions senior managers' ethical leadership relies upon the most to produce employee ethical behavior. Interestingly, this study also contributes by analyzing these relationships in an under-studied country: Malaysia. While ethical leadership research abounds in Western societies (Resick et al., 2011), other world regions have been scarcely explored, and ethical leadership may have different effects in these settings. Malaysia, for example, is a multi-racial society including Malay, Chinese, Indian, and other ethnicities, with important differences in terms of beliefs, religion, ideology, and identity (Weintraub, 2011). It also has certain cultural specificities (i.e., high power distance, Hofstede Center, 1967–2010) that could make the ethical leadership behavior of managers become far more appreciated than in other countries. Thus, this study can offer new insights into how context-sensitive ethical leadership theory (Brown and Treviño, 2006) is in accounting for organizational justice, its specific dimensions, and the ethical behavior of employees.

## Theoretical Background and Hypothesis Development

### Ethical Leadership and Employees' Ethical Behavior

Treviño and Nelson (2004) highlighted that ethical phenomena have existed ever since the dawn of humanity. This is because the human being is, by nature, a moral being, with the capacity to distinguish good from evil, regardless of their birthplace, the historical moment in which he/she lives (21st century) or the religion he/she professes. However, in organizations, having a role model that shows the appropriate path forward is critical in fostering workplace ethical behavior. Even though individuals, as human beings, may question themselves as to “what should they do,” the guidance and direction of leaders play an important role in directing their decisions and actions toward an ethical direction (Trevino et al., 2000). Thus, the challenge of any person in the organization who occupies a relevant management position is to transmit its ethical essence to others in order to build a reputation for ethical leadership. Being seen as an ethical leader implies that others think of you as a person who has ethical qualities, who participates in ethical actions, and makes decisions based on ethical principles. The true ethical leader “walks the talk” and, by doing so, impacts the ethical lives and behaviors of others in the organization (Trevino et al., 2000; Brown and Treviño, 2006).

For the purpose of maintaining effective work situations and ethical standards in organizations, Brown et al. (2005) proposed the concept of “ethical leadership behavior.” This form of leadership recognizes the importance of virtuousness in action, including truthfulness and honesty, and can be defined as “the demonstration of normatively conducted behavior through personal actions and interpersonal relations” (Brown et al., 2005, p. 120). In practice, this means demonstrating aspects such as honesty, integrity, and principled decision-making (Brown et al., 2005).

Brown et al. (2005) highlighted that managers serve as representatives of organizations, meaning they are an important influence for employees in terms of behavior, for social learning (Bandura, 1977) and social exchange (Blau, 1964) motives. According to social learning theory (Bandura, 1977), employees learn appropriate behaviors by paying attention to and emulating the behaviors of attractive, credible role models such as managers. As such, if ethical leadership is practiced by managers, they are likely to influence employee ethical behavior positively (Ruiz et al., 2011; Ruiz-Palomino and Martínez-Cañas, 2011; Ruiz-Palomino and Linuesa-Langreo, 2018; Al Halbusi et al., 2020a). In line with social exchange theory (Blau, 1964), the fair and honest behavior demonstrated by these leaders toward their employees could engender in employees a willingness to trust their leaders (Mayer et al., 2009) and an obligation to reciprocate with positive, ethical behavior (cf., Gouldner, 1960). Thus,

**H1**. Ethical leadership of (senior) managers positively relates to employee ethical behavior.

### The Mediating Role of Organizational Justice

Managers are conferred with the legal power to manage their employees and with taking charge of organizational resources; they are looked upon as carrying out a principal function in their organizations (Loi et al., 2009) and as being in an exclusive position to administer justice (Brown et al., 2005; Ruiz-Palomino et al., 2012). Thus, given that managers are representatives of the organizations (Hou et al., 2018), insofar as employees perceive their managers as ethical, justice, which is implicit in the concept of ethicality (cf., Colquitt, 2001), will be likely perceived as something that is present in their organizations as well. This reasoning become firmer in the case of managers who practice ethical leadership in senior management levels, as these leaders are more likely to be perceived as representative of the organization. In particular, inasmuch as senior managers are perceived as ethical, employees will see that justice is present in the (a) outcomes achieved (distributive justice), (b) procedures realized (procedural justice), (c) relationships established (interpersonal justice), and (d) information received (informational justice) in their organizations (Colquitt, 2001). This is likely to have positive effects on their ethical behavior (Ruiz-Palomino et al., 2012), by activating social exchange processes (El Akremi et al., 2010), as described below.

Rooted in the equity theory developed by Adams (1963) and associated with the equal distribution of outcomes based on the performance of each employee (Burney et al., 2009), distributive justice and its perception in the organization should lead employees to more ethical behaviors. When distributive justice is perceived, the conditions at work should be perceived as more positive (Oshio and Kobayashi, 2009) and reciprocity and social exchange processes should be developed (Gouldner, 1960; Blau, 1964), thus leading employees to respond with positive behaviors toward the leader and organization, such as ethical behavior. In a similar vein, procedural justice, which emphasizes the perceived fairness of the processes followed to make decisions (i.e., Greenberg, 2001; procedures and policies used to determine outcomes or resource distributions, Colquitt, 2001), should lead to more ethical behavior among employees. In effect, a higher level of perceived procedural justice will be accompanied by employees perceiving that they have some voice over the outcome (Lind and Tyler, 1988). According to social exchange processes (Gouldner, 1960; Blau, 1964), this is likely to lead employees to engage in positive behaviors toward the leader and the organization, such as ethical behavior (McCain et al., 2010). As in the procedural and distributive cases, interpersonal justice, defined as the extent to which one perceives that they are being treated with dignity, respect, and fairness (Colquitt, 2001), is also likely to lead to ethical behavior. If employees are well-treated, the norm of reciprocity (Blau, 1964) suggests that these employees will respond to the organization with positive behaviors. Finally, informational justice, which refers to the extent to which communication is developed honestly, fairly (Bies, 2001, and clearly (Colquitt, 2001), is also likely to lead to higher levels of ethical behavior. Employees will see themselves as valued and appreciated and so are likely to respond with positive behavior, in line with reciprocity processes (Gouldner, 1960). For example, in downsizing contexts, informational justice perceptions have been shown to make employees develop trusting attitudes (Brockner et al., 1995), which is one important driver of social exchange processes (Colquitt et al., 2007).

Overall, the extent to which each of these justice dimensions (distributive, procedural, interpersonal, informational) can be shaped by managers' ethical leadership, organizational justice, and each of its different dimensions, is likely to mediate the managerial ethical leadership to employee ethical behavior relationship. Accordingly, we predict the following,

**H2**. Perceived organizational justice and its dimensions of distributive (a), procedural (b), interpersonal (c), and informational (d) justice each mediate the positive relationship between the ethical leadership of (senior) managers and employee ethical behavior.

**Figure 1:**
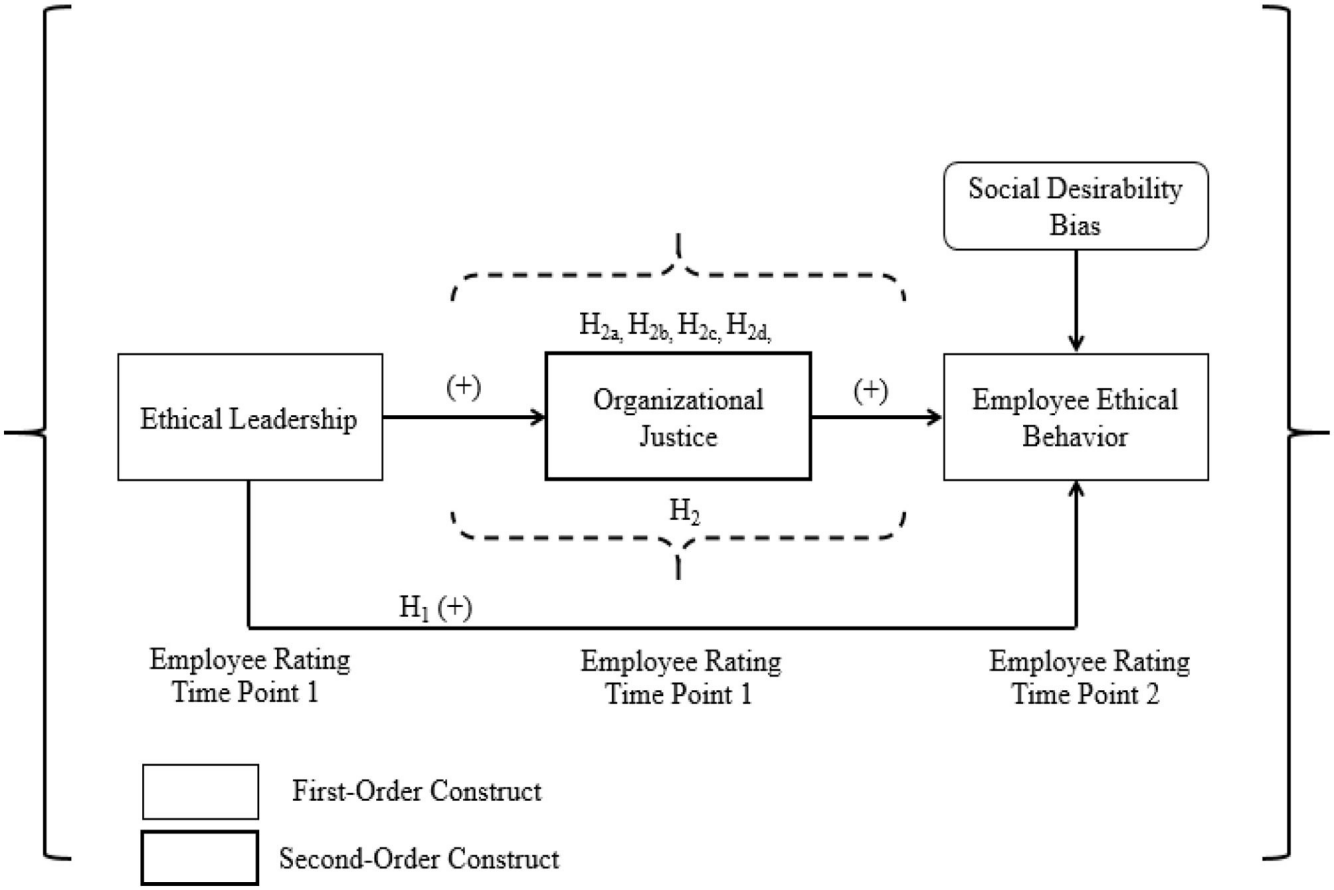
Research model.

Figure 1 shows all the hypotheses proposed in the form of a research model. It includes two main hypotheses (H1, H2) and four sub-hypotheses (H2a, H2b, H2c, H2d).

## Methodology

### Participants and Procedure

After using Brislin's (1980) back-translation procedure, the survey questions were pretested using cognitive interviews with 18 employees from the manufacturing industry. They suggested slight semantic adjustments and confirmed the questionnaire's clarity, readability, comprehension, and suitability. To obtain reliable responses, the questionnaires were distributed to employees with an organizational tenure of at least 6 months. The questionnaires were thus distributed to 350 employees who reported directly to upper/middle managers in 12 manufacturing firms located in Selangor (Malaysia), worked full-time, and had frequent contact with their immediate managers. These questionnaires were distributed in two waves to reduce the occurrence of common method variance (CMV), as recommended (Podsakoff et al., 2012). In the first wave, respondents provided demographic information, together with perceptions of their managers' ethical leadership and organizational justice. In the second wave, after 3 weeks had passed, these respondents rated their own level of ethical behavior. A cover letter was also distributed to respondents, assuring them of total confidentiality and informing them about how important their participation was in this study, which could help reduce social desirability bias (SDB) and CMV (Podsakoff et al., 2003). Both sets of surveys were coded to confirm that the responses could be matched, resulting in 270 valid responses and a 77% response rate. 74.4% of the respondents were men (25.6% women), and the largest group (41.9%) fell in the range between 31 and 40 years. In terms of educational level and job experience, most participants had a bachelor's degree (55.9%), and had been working for the same company for 6–10 years (75%).

### Measurement

The nature of the variables for this study enabled us to differentiate first- (i.e., ethical leadership of senior managers, employee ethical behavior, social desirability bias) and second-order constructs (i.e., organizational justice), captured in Mode A (reflective) constructs, as recommended (Hair et al., 2017). All measures relied on five-point Likert response formats, and apart from organizational justice perceptions (1= “to a small extent” to 5= “to a large extent”), all were based on the respondents' level of agreement on each item surveyed (1 = “strongly disagree”; 5 = “strongly agree”). Table 1 shows the items of these variables.

**Table 1:** Measurement model, loading, construct reliability, and convergent validity.

**1st-Order** ** constructs**	**2nd-Order constructs**	**Items**	**Item description**	**Loading (> 0.5)**	**CA** ** (> 0.7)**	**CR** ** (> 0.7)**	**AVE** ** (> 0.5)**
Ethical Leadership		EL1	My supervisor listens to what employees have to say	0.638	0.910	0.926	0.556
		EL2	My supervisor disciplines employees who violate ethical standards	0.606			
		EL3	My supervisor conducts his/her work in an ethical manner	0.754			
		EL4	My supervisor has the best interests of employees in mind	0.827			
		EL5	My supervisor makes fair decisions	0.733			
		EL6	My supervisor can be trusted	0.753			
		EL7	My supervisor discusses business ethics or values with employees	0.754			
		EL8	My supervisor sets an example of how to do things the right way in terms of ethics	0.812			
		EL9	My supervisor defines success not just by results but also the way that they are obtained	0.756			
		EL10	When making decisions, my supervisor asks… “what is the right thing to do?”	0.796			
Distributive Justice	Rate the following items regarding the outcome(s) (e.g., rewards, pay, promotion) you receive from your organization. To what extent…						
		DJ1	Does your outcome reflect the effort you have put into your work?	0.662	0.750	0.841	0.570
		DJ2	Is your outcome appropriate for the work you have completed?	0.800			
		DJ3	Does your outcome reflect what you have contributed to the organization?	0.795			
		DJ4	Is your outcome justified, given your performance?	0.755			
Procedural Justice	Rate the following items regarding the procedures used to arrive at your outcome(s). To what extent…						
		PJ1	Have you been able to express your views and feelings during those procedures?	0.685	0.707	0.818	0.531
		PJ3	Have those procedures been applied consistently?	0.696			
		PJ5	Have those procedures been based on accurate information?	0.805			
		PJ7	Have those procedures upheld ethical and moral standards?	0.722			
Interpersonal Justice	Rate the following items regarding the authority figure (i.e., superior) who enacted the procedure. To what extent…						
		InterPJ1	Has your superior treated you in a polite manner?	0.853	0.747	0.840	0.572
		InterPJ2	Has your superior treated you with dignity?	0.770			
		InterPJ3	Has your superior treated you with respect?	0.610			
		InterPJ4	Has your superior refrained from inappropriate remarks or comments?	0.771			
Informational Justice	Rate the following items regarding the authority figure (i.e., superior) who enacted the procedure. To what extent…						
		InforJ1	Has your superior been candid in his/her communications with you?	0.807	0.883	0.915	0.682
		InforJ2	Has your superior explained the procedures thoroughly?	0.855			
		InforJ3	Were your superior's explanations regarding the procedures reasonable?	0.838			
		InforJ4	Has your superior communicated details with you in a timely manner?	0.831			
		InforJ5	Has your superior seemed to adapt (his/her) communications to individuals' specific needs?	0.797			
	Organizational Justice	Distributive Justice		0.633	0.879	0.898	0.543
		Procedural Justice		0.752			
		Interpersonal Justice		0.706			
		Informational Justice		0.842			
Ethical behavior		EB1	I take responsibility for my own errors.	0.688	0.880	0.904	0.603
		EB2	I complete time/quality/quantity reports honestly.	0.617			
		EB3	I use company services appropriately and not for personal use.	0.738			
		EB4	I am open about (and do not conceal) my errors.	0.775			
		EB5	I conduct only company business on company time.	0.704			
		EB6	I do not give gifts/favors in exchange for preferential treatment.	0.751			
		EB7	I keep confidential information confidential.	0.767			
		EB8	I take the appropriate amount of time (not longer than necessary) to do a job.	0.677			
		EB9	I report others' violation of company policies and rules.	0.749			
		EB10	I lead my subordinates (or peers) to behave ethically.	0.755			
		EB11	I am careful and do not pilfer company materials and supplies.	0.804			
		EB12	I come to work unless I am sick.	0.769			
SDB		SDB1	There have been occasions when I took advantage of someone (reverse score).	0.582	0.706	0.738	0.506
		SDB2	I sometimes try to get even rather than forgive and forget (reverse score).	0.692			
		SDB3	I've never been annoyed when people expressed ideas very different from my own	0.968			

*CA, Cronbach's Alpha; CR, Composite Reliability; AVE, Average Variance Extracted; SDB, Social Desirability Bias; PJ2, PJ4, PJ6 were dropped due to poor loadings, lower than 0.40*.

#### Ethical Leadership of (Senior) Managers

This variable was assessed using Brown et al.'s (2005) ten-item scale. Responses to all 10 items were combined linearly to form a Mode A first-order composite variable such that higher scores indicated stronger managerial ethical leadership.

#### Organizational Justice

This variable was measured using 20 items from Colquitt's (2001) scale, which refer to “distributive justice,” “procedural justice,” “interpersonal justice,” and “informational justice.” Distributive Justice was measured using four items concentrating on equal payment, promotion, fair recognition, and rewards. A sample item is “Does your outcome reflect the effort you have put into your work?” For Procedural Justice, seven items were used, all of which are intended to measure the extent to which procedures and practices are equally and consistently applied to everyone within the organization. Three items showed poor loadings (far lower than 0.40, Hair et al., 2017), so were dropped. Interpersonal Justice was measured using a four-item scale regarding employees' interaction with their supervisor and referred to whether their supervisor treats them in a polite manner and with dignity. Finally, for Informational Justice, five items were used to measure the extent to which the authority figure enacted the procedures regarding the information being provided. These scales also served to build a Mode A second-order composite (hierarchical common factor), where all justice components represent lower-order constructs that are reflectively measured and are highly correlated (see Becker et al., 2012).

#### Employee Ethical Behavior

When the opportunity to observe others' behaviors is not easy, using reports from others is likely not to be more appropriate than using self-reports (Podsakoff et al., 2012), given that on certain occasions, individuals are the most aware of their own personal behavior (Ruiz-Palomino et al., 2019). Thus, to measure this variable, we slightly adapted a 12-item scale used in previous research (Newstrom and Ruch, 1975; Ferrell and Weaver, 1978) and asked employees to assess their level of agreement on these 12 items, which were a faithful reflection on what ethical behavior is, that is, behavior that rests upon universal moral principles leads to human growth (Ruiz-Palomino et al., 2019) and helps to ensure the good functioning of the organization (Ko et al., 2018). We combined the responses to each of the 12 items linearly to form a Mode A first-order composite variable, such that higher scores indicated stronger employee ethical behavior.

#### Control Variables

Age, gender, job experience, and education served as control variables, as these variables could potentially be related with ethical behavior (O'Fallon and Butterfield, 2005; Craft, 2013). Age, education, and job experience were approached using an ordinal scale anchored at 1 (younger employees, lower education, less job experience) and 5 (older employees, higher education, more job experience). Gender, however, was measured as a dummy variable (0 = male, 1 = female). Finally, because respondents had to evaluate their own ethical behavior, we controlled for social desirability bias (SDB) utilizing three items from Fischer and Fick (1993) to control the extent to which respondents may present themselves to appear better than they actually are. Although some of these SDB items were negatively worded, the three items were coded in such a way that higher scores involved a stronger SDB.

### Common Method Variance (CMV)

In addition to the above-described ex-ante procedural remedies, one *post-hoc* test was conducted to evaluate whether CMV could have biased our findings. It revealed no problems of this type. We ran in AMOS v.24 the common factor tests to compare the difference in standardized weights with and without a common latent factor (CLF); if this difference is >0.2, CMV could be a problem in the data (Podsakoff et al., 2003, 2012). In our study, all the differences were lower than 0.2. Furthermore, the CLF model did not yield a significantly better fit (χ2/df =1.514; CFI = 0.967; RMSEA = 0.0413) compared with the full measurement model (χ2/df = 1.511; CFI = 0.964; RMSEA = 0.0416). This confirms that CMV is not likely to be a serious concern in this study (Podsakoff et al., 2003, 2012).

## Data Analysis and Results

In this research, structural equation modeling (SEM) and, specifically, Partial Least Squares (PLS), via Smart PLS 3.2.8 (Ringle et al., 2018), was used. PLS is a powerful, robust statistical procedure that allows the inclusion of second-order constructs and does not require demanding assumptions about the distribution of the variables (Hair et al., 2017). In addition, PLS provides consistent regression parameters comparable to other structural equation modeling approaches, especially if, as occurs here, a large number of observations and reflective indicators are available (sample size = 270, number of reflective indicators = 52) (Hair et al., 2017). In using PLS, we used 5,000 subsamples to generate standard errors and bootstrap t-statistics with n – 1 degrees of freedom (n is the number of subsamples) to evaluate the statistical significance of the path coefficients (Hair et al., 2017).

### Measurement Model via PLS-SEM

To assess the measurement model, we examined individual item reliability, internal consistency, convergent validity, and discriminant validity (Tables 1, 2). In terms of item reliability, most items exceeded the recommended 0.707 level; otherwise, items generally were above the minimum 0.5 threshold (Hair et al., 2017; see Table 1). To evaluate the internal consistency of the measurement scale, Cronbach's Alpha (CA) and Composite Reliability (CR) were utilized in this study. The ranging of both indices was far above the acceptable level of 0.707, so internal consistency reliability can be supported (Hair et al., 2017). Average Variance Extracted (AVE) was conducted to measure the convergent validity of the study constructs. The evidence of convergent validity was confirmed because the AVE for constructs exceeded the threshold of 0.50, as recommended (Hair et al., 2017). Discriminant validity was also confirmed by HTMT inference and Fornell-Larcker criterions; HTMT values were significantly different from 1, and the square roots of AVE for each variable were greater than the correlation of each variable with the others (Hair et al., 2017; see Table 2).

**Table 2:** Descriptive statistics, correlation matrix, and discriminant validity (AVE in bold and HTMT in italics).

**Constructs**	**Mean**	**SD**	**1**	**2**	**3**	**4**	**5**	**6**	**7**	**8**
1. Ethical leadership	4.160	0.550	**0.826**	*0.673* * [0.603;0.737]*	*0.633* * [0.549;0.719]*	*0.197* * [0.157;0.254]*	*0.103* * [0.089;0.150]*	*0.071* * [0.061;0.150]*	*0.060* * [0.052;0.095]*	*0.084* * [0.068;0.121]*
2. Organizational justice	3.930	0.410	0.603	**0.737**	*0.736* * [0.682;0.787]*	*0.175* * [0.156;0.239]*	*0.152* * [0.136;0.223]*	*0.139* * [0.110;0.227]*	*0.078* * [0.074;0.128]*	*0.175* * [0.155;0.217]*
3. Ethical behavior	4.090	0.510	0.580	0.659	**0.777**	*0.180* * [0.152;0.247]*	*0.179* * [0.121;0.273]*	*0.167* * [0.125;0.252]*	*0.104* * [0.096;0.139]*	*0.075* * [0.062;0.125]*
4. Social desirability	2.821	0.767	0.049	0.074	0.075	**0.711**	0.087 *[0.063;0.132]*	2.811 *[0.005;0.153]*	*0.118* * [0.073;0.188]*	*0.106* * [0.062;0.166]*
5. Gender	n.a	n.a	0.010	0.070	0.168	0.047	**n.a**	*0.018[0.005;0.134]*	*0.015 [0.002;0.103]*	*0.050* *[0.005;0.119]*
6. Age	n.a	n.a	0.020	−0.100	−0.1530	0.113	−0.020	**n.a**	*0.268* * [0.206;0.331]*	*0.660* * [0.623;0.698]*
7. Education	n.a	n.a	−0.060	−0.110	−0.100	0.064	0.020	0.186	**n.a**	*0.166* * [0.110;0.225]*
8. Job experience	n.a	n.a	0.030	−0.010	−0.050	0.059	−0.010	0.728	0.109	**n.a**

*SD, Standard Deviation; n.a., not applicable. Off-diagonal elements below the diagonal are correlations among the constructs, where values between 0.12 and 0.15 are significant at p < 0.05 (two-tailed test)), and values above 0.15 are significant at p < 0.01 (two-tailed test). Off-diagonal elements above the diagonal are the heterotrait-monotrait ratios of correlations (HTMT), and their respective confidence intervals at the 95% confidence level*.

### Hypothesis Testing

**Figure 2:**
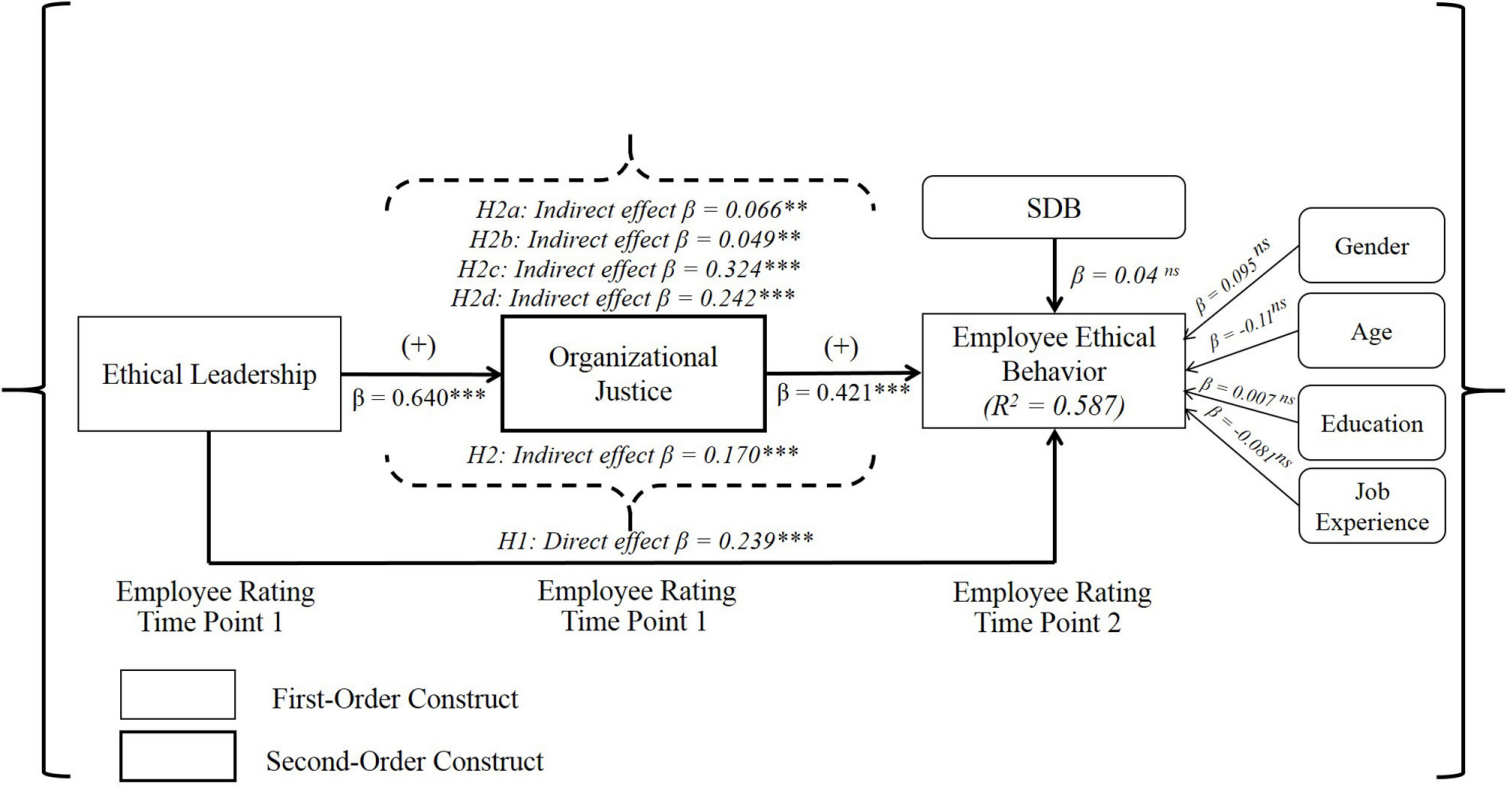
Structural model: hypothesis testing. SDB, Social Desirability Bias; ***p < 0.001, **p < 0.01, ns, not significant.

None of the demographic variables (i.e., gender, age, education, and job experience) showed a significant impact on employee ethical behavior (Figure 2). The influence of SDB was not significant either (β = 0.04 ns, Figure 2), thus showing no influence of this bias in this study. Regarding hypothesis testing, the ethical leadership of (senior) managers was observed to influence employee ethical behavior positively (β = 0.239, *p* < 0.001; Table 3, Figure 2), in support of H1. Our findings also reveal support for our prediction in H2 and its sub-hypotheses. First, a significant indirect effect of the ethical leadership of (senior) managers on employee ethical behavior through organizational justice exists (indirect effect= 0.170, *p* < 0.001; Table 3, Figure 2), in support of H2; in terms of effect size (*f*^2^), this effect is large (*f*^2^ = 0.550; Table 3, Figure 2). Second, the results also give wide support to the sub-hypotheses derived from H2. As Table 3 shows, distributive justice mediates between managerial ethical leadership and employee ethical behavior (indirect effect = 0.066, *p* < 0.01), in support of H2a. Procedural justice mediates in this relationship as the indirect effect is positive and significant (indirect effect = 0.049, *p* < 0.01). Table 3 also supports mediation for interpersonal justice, by revealing a significant, positive indirect effect of ethical leadership on employee ethical behavior (indirect effect = 0.324, *p* < 0.001). Finally, informational justice was also a significant mediator in the relationship (indirect effect = 0.242, *p* < 0.001, Table 3). Thus, each of the dimensions of organizational justice (distributive, procedural, interpersonal, and informational) are significant mediators, and H2a to H2d can be supported (Table 3, Figure 2). Importantly, however, some justice dimensions were more important mediators than others. Whereas, interpersonal and informational justice showed the strongest size effects (*f*^2^ = 0.116 for interpersonal justice; *f*^2^ = 0.063 for informational justice), procedural and distributive justice showed the weakest effect sizes (small to moderate for procedural justice, *f*^2^ = 0.045, and small for distributive justice, *f*^2^ = 0.017).

**Table 3:** Structural path analysis results: direct and mediation effects.

**Direct effect:**						**95% Bias and corrected confidence interval**					
**Hypothesis**	**Relationship**	**Beta**	**SD**	***t*-value**	***p*-value**	**[Lower level;**	**Upper level]**	**Hypothesis**	***R^**2**^***	***Q^**2**^_***EB***_***	
H1	EL->EB	0.239	0.070	3.437	0.000	[0.124	0.339]	Supported	0.357	0.222	
**Mediation Effect:**											
						**95% Bias and corrected confidence interval**					
**Hypothesis**	**Relationship**	**Indirect effect**	**SD**	***t*****-value**	***p*****-value**	**[Lower level;**	**Upper level]**	**Hypothesis**	***R**^**2**^*	***f**^**2**^*	**$Q_{OJ}^{2}$**
H2	EL-> OJ->EB	0.170	0.045	3.817	0.000	[0.163	0.260]	Supported	0.587	0.550	0.161
H2a	EL-> DJ->EB	0.066	0.025	2.602	0.005	[0.023	0.104]	Supported	0.374	0.017	0.037
H2b	EL-> PJ->EB	0.049	0.018	2.759	0.003	[0.020	0.076]	Supported	0.402	0.045	0.129
H2c	EL-> InterPJ->EB	0.324	0.049	6.565	0.000	[0.246	0.406]	Supported	0.473	0.116	0.134
H2d	EL-> InforJ->EB	0.242	0.054	4.455	0.000	[0.151	0.322]	Supported	0.420	0.063	0.126

*EL->EB, Ethical Leadership-> Ethical Behavior; SD, Standard Deviation. EL, Ethical Leadership; DJ, Distributive Justice; PJ, Procedural Justice; InterPJ, Interpersonal Justice; InforJ, Informational Justice; OJ, Organizational Justice.f^2^ = (R^2^ included – R^2^ excluded)/(1 – R^2^ included); effect sizes of f^2^ ≥ 0.02, ≥ 0.15, and ≥ 0.35 are small, moderate, and large, respectively (Cohen, 1988)*.

In terms of explanatory power, the model explains moderate to substantial variance (*R*^2^ = 0.587) of employee ethical behavior (Hair et al., 2017). The Stone-Geisser blindfolding sample reuse technique revealed *Q*^2^*-*values larger than zero, thus indicating good predictive power for overall organizational justice (*Q*^2^ = 0.161) and each of its dimensions (*Q*^2^_distributive_ = 0.037; *Q*^2^_procedural_ = 0.129, *Q*^2^_interpersonal_ = 0.134, *Q*^2^_informational_ = 0.126), and employee ethical behavior (*Q*^2^ = 0.222) (Hair et al., 2017). Finally, in terms of overall goodness-of-fit (GoF), the SRMR index (standardized root means square residual) offered a value of 0.041, which is far below the 0.08 cut-off (Henseler, 2017); the SRMR's 95% bootstrap quantile was 0.054 and therefore higher than the SRMR value, indicating that the model has a good fit (Hair et al., 2017). The discrepancy indices dULS (unweighted least squares discrepancy) and dG (geodesic discrepancy) were also below the bootstrap-based 95% percentile (dULS = 1.421 < HI 95 of dULS = 2.521; dG = 0.562 < HI 95 of dG = 0.989), thus confirming good model fit and indicating that the model tested in this study is likely to be valid (Henseler, 2017).

## Discussion and Conclusion

This study investigated the relationship between ethical leadership of senior managers and employee ethical behavior by analyzing organizational justice perceptions as a mediator. The results obtained lead to two main conclusions. First, managerial ethical leadership is a fueling factor in obtaining high levels of ethical behavior among employees. Second, the influence of ethical senior managers on employee ethical behavior rests mainly upon making employees perceive (a) overall organizational justice and (b) each of its dimensions: distributive, procedural, interpersonal, or informational justice. Importantly, each of the different forms of justice mediates in the relationship between the ethical leadership of these managers and employee ethical behavior, with interpersonal and informational the justice dimensions with the greatest mediation effects.

### Theoretical Implications

This study makes an important contribution to the ethical leadership and ethical behavior literature. Prior studies have found ethical leadership to have a positive effect on employees' ethical behavior (e.g., Mayer et al., 2009; Ruiz-Palomino and Linuesa-Langreo, 2018), but the mechanisms connecting the ethical leadership of senior managers to employees' ethical behavior are still not well-established (Treviño et al., 2014). This study fills this gap to some extent and puts forward organizational justice perceptions as a possible mechanism between ethical leadership of senior managers and employee ethical behavior. In particular, an interesting advancement of the literature is showing the strong connection between the ethical leadership phenomenon and the development of justice perceptions regarding the procedures (i.e., procedural justice), the outcomes received (distributive justice), the relationships that are held with the manager (interpersonal justice), and the information received within the organization (informational justice), as well as the differentiated mediation role of each of these justice dimensions in the ethical leadership–employee ethical behavior relationship. Thus, for example, the interpersonal and informational dimensions of organizational justice have the strongest individual mediating roles in this relationship. This is in line with previous research showing that, compared to procedural and distributive justice, interpersonal and informational justice tend to have stronger effects on employee attitudes and behaviors in general (Williams et al., 2002; Ambrose and Schminke, 2003). This is probably because procedural and distributive justice, which are about the fairness of decision-making processes and its outcomes, are linked to “resource exchange” contexts, whereas interpersonal and informational justice involve “encounters” (Bies, 2005). The former are more infrequent than the latter, as these occur between managers and employees, suggesting that interpersonal and informational justice have a stronger “day-to-day” significance than procedural and distributive justice. They could therefore exert a stronger influence on employees' workplace behavior, including ethical behavior.

Another important implication is that the current investigation brings ethical leadership, organizational justice—including all its dimensions—and employee ethical behavior together in a non-Western context (Malaysia). Existing ethical leadership research has typically over-emphasized Western countries (Resick et al., 2011), but new investigations focusing on other cultural contexts are necessary to generalize the robustness of the theory underlying the ethical leadership phenomenon and its positive outcomes in the workplace. This study is set in a multi-racial country (Malaya, Chinese, Indian; Weintraub, 2011), and reveals the influence of ethical leadership on employee ethical behavior via the way it shapes organizational justice. This represents a positive development regarding the generalization of the direct and mediated relationship between ethical leadership and employee ethical behavior across distinct ethnicities. Malaysia is known for its extremely “high power” distance compared to the US and other Western cultural contexts (Hofstede Center, 1967–2010). Malaysia is also a country where Islam is predominant (Weintraub, 2011), a religion on which ethical leadership research is still not abundant (Metwally et al., 2019). Thus, by demonstrating the positive effect of ethical leadership on employee ethical behavior via organizational justice, and the stronger mediation size effect of interpersonal and informational justice dimensions in such a relationship, this study helps give robustness to the theory of ethical leadership and its outcomes across national cultural geographies.

### Managerial Implications

From a managerial point of view, this study has a number of important implications. First, in identifying that ethical leadership of senior managers is important in fostering ethical behavior in the workplace, we make an important contribution to managerial practice. All efforts to understand the mechanisms that help such behavior to emerge are interesting from a managerial perspective due to the important benefits generally associated with having ethical employees. Ethical employees are more willing to put in extra effort to finish their work on time and follow the ethical standards indicated by their employer; they try to be a representative of the company in terms of public relations even when they are not at work, and make efforts to promote the organization's development (Treviño et al., 2014). Therefore, given that the practice of ethical leadership at all managerial levels can serve to spread ethical behavior within the organization, we believe that Human Resource (HR) managers should devote time and attention to hiring and training candidates for managerial positions. In other words, considering the benefits of ethical leadership in increasing employee ethical behavior, HR managers need to try their best to recruit, select, and train suitable persons who are willing to practice and develop an ethical leadership approach. Organizations should use a variety of assessment tools such as focus groups, in-basket exercises, structured interviews, and business games that focus on or involve relevant ethical issues to evaluate whether candidates have core traits of ethical leaders (e.g., honesty, fairness, caring, Brown et al., 2005). Organizations can also design training programs oriented toward developing managers' ethical leadership behaviors in their day-to-day activities. These could include strategies to support trainees (specifically, current or future managers) on how to make ethical decisions or act ethically, communicate about ethics and values, and/or set up ethical examples for others (e.g., subordinates or team members) in their respective organizations.

Second, although the practice of ethical leadership by managers impacts the ethical behavior of employees directly, there is also an indirect influence through enhancing employees' organizational justice perceptions. Thus, organizational justice becomes an important mechanism through which ethical managers prompt ethical behavior among their employees. Managers should thus become aware that all efforts directed to make employees perceive that justice is present in the outcomes achieved, the procedures realized, the supervisor–employee relationships that are established, and the information that is received can have a significant role in fostering ethical behavior in the workplace. Of special importance is that managers can be trained in providing their followers with proper rationales of their decisions and on treating them with high levels of dignity and respect (interpersonal justice). Also, managers could increase their followers' employee ethical behavior by ensuring that smooth top–down communication channels become established, which can help employees to obtain first-hand information about what is of relevance to their interests (informational justice).

### Limitations and Future Research

The first limitation of the current study concerns our cross-sectional data design, which makes it difficult to provide definitive conclusions about causality. We recommend that future research uses experimental or longitudinal designs that will help reinforce the causality findings of the current study. In addition, our conclusions are limited by the cultural context of the study (Malaysia), so we recommend that future studies replicate our study in other cultures to improve the study's external validity and generalizability.

The second limitation relates to the data we used. In this study, our data came from a single source. Although we conducted two waves of surveys (Podsakoff et al., 2012), we cannot rule out CMV completely, and the rigor of our empirical findings could have been negatively affected, albeit minimally. However, the *post-hoc* test conducted (i.e., common latent factor, Podsakoff et al., 2003) revealed no serious concerns regarding CMB. In addition, because data was not collected from multiple sources, we could not rule out social desirability response bias completely. However, we included in our analysis a variable that tried to measure the extent to which respondents were more prone to answer the questionnaire in a socially responsible manner, so our findings were controlled by this potential bias, as recommended (Randall and Fernandes, 2012). Future studies, however, could collect data from multiple sources. Although ethical behavior is not easy to observe (Randall and Fernandes, 2012), future researchers could obtain more reliable data by having different informants (Treviño et al., 2006): close workmates rather than supervisors, as the former will likely offer more reliable information. Also, using laboratory research designs such as in-basket exercises and computer simulations could help to obtain highly reliable information (Treviño et al., 2006); these techniques allow respondents to be presented with multiple scenarios, where only some have ethical implications, so respondents are less likely to note that their ethical decision-making or behavior is being measured.

Third, ethical leadership and organizational justice perceptions appear to be related to employee ethical behavior, this association is likely to be dependent on certain boundaries. The workplace environment has an enormous influence on ethical decision-making processes; however, individual differences also play an important role (O'Fallon and Butterfield, 2005; Craft, 2013). In this regard, recent research exploring cognitive processing of moral cues suggests that employees differ in the extent to which they pay attention to moral issues and therefore differ in the moral attentiveness they show in their day-to-day activities (Reynolds, 2008). Thus, the moral cues offered by either ethical leadership or the fair outcomes, processes, interactions, and communication perceived (i.e., organizational justice) may be interpreted or captured in a different way by individuals who have higher rather than lower moral attentiveness. With greater moral attentiveness, the moral clues perceived thanks to the presence of ethical leaders and organizational justice may be more salient. This offers an interesting future line of research to advance on how ethical leadership can become more effective at work.

Fourth, there are major variations in Malaysia in terms of the beliefs, spirituality and religion of each ethnic group (Weintraub, 2011). Future researchers could thus implement studies that could evaluate if these differences in beliefs, religiosity, and spirituality may affect our findings (Whetten, 2009; Ribberink et al., 2018). Importantly, since this study was conducted in the context of an “Islamic” perspective, variables such as “Islamic work ethic” and “shariah compliance” may constitute critical conditional variables to be explored.

Finally, in this study, we did not examine other leadership styles (e.g., transformational leadership, transactional leadership) and did not devote efforts to assessing their influence on employee ethical behavior. However, some partial overlap can exist between ethical leadership and other leadership styles (Brown et al., 2005; Brown and Treviño, 2006). For instance, Brown et al. (2005) suggest that ethical leadership could include a series of transformational behaviors, such as engaging in ethical decision-making and showing genuine concern for subordinates' well-being. Managers may also display transactional behaviors to promote employee ethical behavior, and these behaviors may include aspects such as communicating ethical standards or (punishing) rewarding (un)ethical behavior, which is very characteristic of ethical leaders (Brown et al., 2005). Therefore, future inquiries could explore differences vs. similarities of the ethical leadership approach compared to other leadership approaches in explaining employee ethical behavior via organizational justice.

## Data Availability Statement

The datasets generated for this study are available on request to the corresponding author.

## Author Contributions

HA designed the study, conceived the idea and collected the data, carried out the data analysis, and contributed to the writing. PR-P, PJ-E, and SG-B contributed to the writing and the whole revision process equally.

## Conflict of Interest

The authors declare that the research was conducted in the absence of any commercial or financial relationships that could be construed as a potential conflict of interest.
